# Evaluating the discrepancies between evidence-based and community standard practices in the endoscopic examination of Barrett’s esophagus: a nationwide survey in Japan

**DOI:** 10.1007/s10388-025-01127-6

**Published:** 2025-04-19

**Authors:** Yugo Iwaya, Katsunori Iijima, Takuto Hikichi, Yuji Amano, Masaki Endo, Kenichi Goda, Tomoaki Suga, Makoto Yamasaki, Masashi Kawamura, Fumisato Sasaki, Koji Tanaka, Ken Namikawa, Manabu Muto, Hiroya Takeuchi, Ryu Ishihara

**Affiliations:** 1 Department of Medicine, Division of Gastroenterology and Hepatology, Shinshu University School of Medicine, Asahi 3-1-1, Matsumoto, Nagano 390-8621 Japan; 2 Department of Gastroenterology, Akita University Graduate School of Medicine, Akita, Japan; 3 Department of Endoscopy, Fukushima Medical University Hospital, Fukushima, Japan; 4 Urawa Gastrointestinal Endoscopy Clinic, Saitama, Japan; 5 Kaiunbashi Endoscopy Clinic, Iwate, Japan; 6 Gastrointestinal Endoscopy Center, Dokkyo Medical University Hospital, Tochigi, Japan; 7 Department of Gastroenterology, Japanese Red Cross Society, Suwa Hospital, Nagano, Japan; 8 Department of Surgery, Kansai Medical University, Osaka, Japan; 9 Department of Gastroenterology, Sendai City Hospital, Sendai, Japan; 10 Digestive and Lifestyle Diseases, Kagoshima University Graduate School of Medical and Dental Sciences, Kagoshima, Japan; 11 Department of Gastroenterological Surgery, Osaka University Graduate School of Medicine, Osaka, Japan; 12 Department of Gastroenterology, Cancer Institute Hospital of Japanese Foundation for Cancer Research, Tokyo, Japan; 13 Department of Gastroenterology, Landspítali - The National University Hospital of Iceland, Reykjavík, Iceland; 14 Department of Medical Oncology, Graduate School of Medicine, Kyoto University, Kyoto, Japan; 15 Department of Surgery, Hamamatsu University School of Medicine, Shizuoka, Japan; 16 Department of Gastrointestinal Oncology, Osaka International Cancer Institute, Osaka, Japan

**Keywords:** Biopsy, Esophageal adenocarcinoma, Evidence-based medicine, Gastrointestinal endoscopy, Guideline adherence

## Abstract

**Background:**

Barrett’s esophagus (BE) is a known precursor of esophageal adenocarcinoma (EAC). EAC is comparatively rare in Japan compared to Western countries, where BE management guidelines have been well established based on robust evidence. This study evaluated for gaps between evidence-based medicine (EBM) and real-world clinical practice for BE management in Japan and examined endoscopist adherence to Japanese and Western guidelines.

**Methods:**

A nationwide survey consisting of 19 questions was conducted among Japanese endoscopists to assess their diagnostic and surveillance practices for BE. Descriptive statistics and multivariate logistic regression analysis were employed to interpret key data.

**Results:**

Responses from 804 endoscopists revealed significant differences between Western guidelines and Japanese practices. Local adherence to standardized inspection times was 7.6%, and 30.7% of endoscopists used the Prague classification. Biopsies for BE diagnosis and random biopsies following the Seattle protocol were rarely performed. For long-segment BE, 51.4% of respondents reported using magnifying endoscopy. Regarding ultra-short-segment BE (USSBE), opinions were divided on whether it should be diagnosed as BE and if patients should be informed of its diagnosis. Approximately 40% of respondents advocated annual surveillance for USSBE, with a general tendency to recommend closer follow-up regardless of BE length as compared with Western guidelines.

**Conclusions:**

This survey highlighted several incongruities between EBM and real-world practices for BE, as well as differences between Western and Japanese approaches. Bridging these gaps will require generating more Japan-specific evidence, refining guidelines, and then promoting their dissemination to harmonize best BE practices with international standards and Japanese clinical settings.

**Supplementary Information:**

The online version contains supplementary material available at 10.1007/s10388-025-01127-6.

## Introduction

The incidence of esophageal adenocarcinoma (EAC) is gradually increasing in Japan [1, 2], although it remains significantly less prevalent than in Western countries, where extensive research has established evidence-based guidelines for Barrett’s esophagus (BE) management [3–6]. In Japan, the rarity of EAC has resulted in limited domestic evidence on which to base clinical decision-making. Thus, there remains uncertainty on the applicability of Western guidelines in the Japanese context.

Prior surveys on esophageal squamous cell carcinoma (ESCC) have identified substantial discrepancies between evidence-based medicine (EBM) standards and local real-world practices in Japan, emphasizing the need to address these gaps [7]. Accordingly, we conducted a nationwide survey to investigate the differences between EBM and community standards in BE management and assess endoscopist adherence to Western and Japanese guidelines. This study aimed to clarify how local endoscopists approach BE management under multiple guideline frameworks in the real-world setting.

## Methods

### Survey

An online survey was conducted among Japanese endoscopists who performed upper gastrointestinal endoscopy at least once per week. The multiple-choice survey consisted of 19 questions in Japanese, which were developed, reviewed, and tested by the core study team members (YI, KI, TH, and RI) to ensure clarity and relevance. The questionnaire focused on clinical practices related to BE management and was built using Google Forms (Google, CA, USA), with an estimated time of 4–5 min required to complete all questions.

Invitations to participate in the survey were disseminated through email lists provided by the Japan Esophageal Society (JES), the FIGHT-Japan Study Group, and the Endoscopic Atlas Group, as well as individual (YI, KI, TH, and RI) mailing lists. The primary goal of this survey was to collect responses from a broad range of actively practicing endoscopists in Japan. The email lists are widely subscribed to by endoscopists across Japan and include not only esophageal specialists but also general gastroenterologists and other endoscopists, thereby ensuring a diverse range of perspectives on BE management. The initial invitation was sent on September 6, 2024, with a reminder email sent on September 20, 2024. The survey period closed on September 30, 2024. Responses were included in this analysis if the respondent performed at least one endoscopic examination per week.

The interpretations of responses were reviewed from multiple perspectives by team members affiliated with various types of institutions and specialties, including university hospitals, cancer specialty hospitals, general hospitals, clinics, and surgical departments. Given the study’s non-interventional nature and design and the fact that no patient data or personal information were collected, ethical approval was judged as unnecessary by our institutional ethics committee.

### Statistics

Descriptive statistical analysis was performed, with results expressed as numbers and frequencies (%). Additional analyses were conducted for questions 3, 4, and 9, for which substantial variability in the responses was observed. Multiple logistic regression was applied for multivariate analysis to identify independent factors associated with the outcome. All clinically relevant variables were initially entered into the model. We then used a backward stepwise elimination procedure based on Wald tests, whereby variables with *p* ≥ 0.05 were sequentially removed. This process was repeated until all remaining variables satisfied *p* < 0.05. The variance inflation factor was examined to assess multicollinearity, and the final model’s goodness of fit was evaluated via deviance and the likelihood ratio test. Adjusted odds ratios and 95% confidence intervals were calculated from the final model. All *p* values were two-sided, with *p* < 0.05 considered statistically significant. Statistical analyses were performed using EZR software (Saitama Medical Center, Jichi Medical University, Saitama, Japan).

## Results

### Survey respondents (including questions 1 and 2)

A total of 821 physicians completed surveys. Seventeen were excluded from the study due to fewer than one endoscopic examination per week, leaving a final sample of 804 endoscopists for analysis. The demographics and practice settings of the included respondents are summarized in Table 1. A total of 231 (28.7%) endoscopists were affiliated with university hospitals, 45 (5.6%) with cancer specialty hospitals, and 322 (40.0%) with general hospitals. An additional, 163 (20.3%) respondents worked in clinics, with 43 (5.3%) reporting other practice settings.

**Table 1:** Profile of the survey respondents

Age, *n* (%)	
≤ 30 years	22 (2.7%)
31–40 years	181 (22.5%)
41–50 years	275 (34.2%)
51–60 years	194 (24.1%)
61–70 years	110 (13.7%)
≥ 71 years	22 (2.7%)
Sex, *n* (%)	
Female	115 (14.3%)
Male	689 (85.7%)
Year of medical license acquisition, *n* (%)	
≤ 1970	7 (0.9%)
1971–1980	35 (4.4%)
1981–1990	112 (13.9%)
1991–2000	201 (25.0%)
2001–2010	266 (33.1%)
2011–2023	183 (22.8%)
Work location area, *n* (%)	
Hokkaido	10 (1.2%)
Tohoku	205 (25.5%)
Kanto	208 (25.9%)
Chubu	122 (15.2%)
Kinki	93 (11.6%)
Chugoku-Shikoku	91 (11.3%)
Kyushu	75 (9.3%)
Specialty, *n* (%)	
Gastroenterology	661 (82.2%)
Gastroenterological surgery	107 (13.3%)
Other	36 (4.5%)
Proportion of endoscopic practice within total workload, *n* (%)	
0–24%	202 (25.1%)
25–49%	210 (26.1%)
50–74%	237 (29.5%)
75–100%	155 (19.3%)
Member of the Japan Esophageal Society, *n* (%)	
Yes	246 (30.6%)
No	558 (69.4%)
Japanese Esophageal Society esophagologist certification, *n* (%)	
Yes	117 (14.6%)
No	687 (85.4%)
Board-certified fellow or trainer of the Japan Gastroenterological Endoscopy Society, *n* (%)	
Yes	657 (81.7%)
No	147 (18.3%)
Primary work facility, *n* (%)	
University hospital	231 (28.7%)
Cancer specialty hospital	45 (5.6%)
Other hospital	322 (40.0%)
Clinic (1–19 beds)	11 (1.4%)
Clinic (without beds)	152 (18.9%)
Other	43 (5.3%)
Main purpose of endoscopic examinations conducted, *n* (%)	
Regular medical practice	664 (82.3%)
Health checkups and screenings	135 (16.8%)
Other	5 (0.6%)
Number of ESCC and EAC patients examined by endoscopy annually at main work facility, *n* (%)	
< 100 patients	637 (79.2%)
≥ 100 patients	167 (20.8%)
Q1. Approximate number of weekly upper gastrointestinal endoscopy examinations, *n* (%)	
1–5 patients	99 (12.3%)
6–10 patients	130 (16.2%)
11–15 patients	135 (16.8%)
16–20 patients	139 (17.3%)
21–25 patients	113 (14.1%)
26–30 patients	69 (8.6%)
≥ 31 patients	119 (14.8%)
Q2. Most frequently used endoscopy system, *n* (%)	
Olympus	645 (80.2%)
Fujifilm	159 (19.8%)
Pentax	0 (0.0%)

*ESCC* esophageal squamous cell carcinoma, *EAC* esophageal adenocarcinoma

The survey was distributed via individual email addresses, and recipients were encouraged to broadly forward the survey link. Thus, it was not possible to determine the precise number of invitations sent or the response rate.

### Observation time and documentation of BE (questions 3 and 4)

As shown in Table 2, the majority of respondents either did not allocate sufficient time or were not conscious of observation time when examining patients with long-segment BE (LSBE). We observed that 7.6% of endoscopists made a conscious effort to spend at least one minute per centimeter when observing LSBE. Multivariate analysis revealed that membership in the JES and having an annual esophageal cancer (ESCC and EAC) volume of 100 patients or more at the primary workplace were significant independent factors contributing to spending at least one minute per centimeter during LSBE observation (Supplementary Table S1). Furthermore, 30.7% of endoscopists reported using the Prague classification [7] when documenting the length of BE. Multivariate analysis on the use of the Prague classification showed that specialty, membership in the JES, and the number of ESCC and EAC patients examined by endoscopy annually at the main work facility were significant contributing factors (Supplementary Table S2).

**Table 2:** Survey questions and responses from 804 endoscopists (translated from Japanese)

Q3: When performing endoscopy on patients with long-segment Barrett’s esophagus (LSBE) of 3 cm or more in maximum length, how much time do you spend based on the length of the Barrett’s segment?—*n* (%)	
≥ 1 min per 1 cm	61 (7.6%)
< 1 min per 1 cm	388 (48.2%)
Do not pay much attention	355 (44.2%)
Q4: When documenting the length of Barrett’s esophagus, do you use the Prague classification?—*n* (%)	
Yes	247 (30.7%)
No	444 (55.2%)
Do not know about Prague classification	113 (14.1%)
Q5: When performing endoscopy under sedation, how do you endoscopically determine the esophagogastric junction (EGJ)?—*n* (%)	
Distal end of the palisade vessels	381 (47.4%)
Proximal end of the gastric folds	105 (13.1%)
Both	267(33.2%)
Not sure	51 (6.3%)
Q6: When performing endoscopy without sedation, how do you endoscopically determine the esophagogastric junction (EGJ)?—*n* (%)	
Distal end of the palisade vessels	498 (61.9%)
Proximal end of the gastric folds	75 (9.3%)
Both	219 (27.2%)
Not sure	12 (1.5%)
Q7: Do you take biopsies to confirm intestinal metaplasia for the diagnosis of Barrett’s esophagus?—*n* (%)	
Yes	48 (6.0%)
No	756 (94.0%)
Q8: Do you perform random biopsies as per the Seattle protocol used in Western countries to detect early-stage cancer in Barrett’s esophagus?—*n* (%)	
Yes	6 (0.7%)
No	748 (93.0%)
Depends on the length of Barrett’s esophagus	50 (6.2%)
Q9: When performing endoscopy on a patient with known long-segment Barrett’s esophagus (LSBE), which endoscope do you most frequently use?—*n* (%)	
Magnifying endoscope	413 (51.4%)
Non-magnifying endoscope	227 (28.2%)
Ultra-thin endoscope	164 (20.4%)
Q10: When examining patients with long-segment Barrett’s esophagus (LSBE), which mode do you primarily use for observation? (multiple answers allowed)—*n* (%)	
NBI or BLI	755 (93.9%)
TXI or LCI (without NBI or BLI)	18 (2.2%)
WLI (without NBI, BLI, TXI, or LCI)	31 (3.9%)
Q11: When observing the distal end of the esophagus, at what length of columnar epithelium do you diagnose Barrett’s esophagus (and document in the report)?—*n* (%)	
All lengths, including < 1 cm	431 (53.6%)
≥ 1 cm	298 (37.1%)
≥ 2 cm	32 (4.0%)
≥ 3 cm	18 (2.2%)
Not sure	25 (3.1%)
Q12: For those who selected an option other than “All lengths, including < 1 cm” or”Not sure” in Q11, what are your reasons for not diagnosing columnar epithelium of less than 1 cm as Barrett’s esophagus (and not documenting it in the report)? (multiple answers allowed)—*n* (%)	
Too many patients meet the criteria	216/348 (62.1%)
Low cancer risk for < 1 cm	131/348 (37.6%)
< 1 cm is considered normal	121/348 (34.8%)
Avoid causing unnecessary anxiety	83/348 (23.9%)
Other	3/348 (0.9%)
Q13: When Barrett’s esophagus is observed during a screening endoscopy, at what length do you inform the patient of its presence?—*n* (%)	
All lengths, including < 1 cm	247 (30.7%)
≥ 1 cm	324 (40.3%)
≥ 2 cm	73 (9.1%)
≥ 3 cm	113 (14.1%)
Not sure	47 (5.8%)
Q14: When ultra-short segment Barrett’s esophagus (USSBE) of less than 1 cm in maximum length is observed during a screening endoscopy, how frequently do you perform surveillance?—*n* (%)	
Do not recommend special surveillance	348 (43.3%)
Every 6 months	1 (0.1%)
Every 1 year	339 (42.2%)
Every 2 years	80 (10.0%)
Every 3–5 years	24 (3.0%)
Not sure	12 (1.5%)
Q15: When short-segment Barrett’s esophagus (SSBE) of 1 cm or more but less than 3 cm in maximum length is observed during a screening endoscopy, how frequently do you perform surveillance?—*n* (%)	
Do not recommend special surveillance	151 (18.8%)
Every 6 months	17 (2.1%)
Every 1 year	528 (65.7%)
Every 2 years	82 (10.2%)
Every 3–5 years	20 (2.5%)
Not sure	6 (0.7%)
Q16: When long-segment Barrett’s esophagus (LSBE) of 3 cm or more in maximum length is observed during a screening endoscopy, how frequently do you perform surveillance? – *n* (%)	
Do not recommend special surveillance	21 (2.6%)
Every 6 months	148 (18.4%)
Every 1 year	616 (76.6%)
Every 2 years	9 (1.1%)
Every 3–5 years	0 (0%)
Not sure	10 (1.2%)
Q17: When diagnosing Barrett’s esophagus, do you administer PPI/P-CAB treatment? (multiple answers allowed)—*n* (%)	
Administer if symptomatic	601 (74.8%)
Administer if reflux esophagitis is present	425 (52.9%)
Administer regardless of Barrett’s length	9 (1.1%)
Administer if Barrett’s length is ≥ 1 cm	8 (1.0%)
Administer if Barrett’s length is ≥ 3 cm	51 (6.3%)
Do not administer in principle	74 (9.2%)
Not sure	6 (0.7%)
Q18: Do you administer PPI/P-CAB treatment to patients with reflux esophagitis after subtotal esophagectomy? (multiple answers allowed)—*n* (%)	
Administer if symptomatic	626 (77.9%)
Administer if LA grade A/B	127 (15.8%)
Administer if LA grade C/D	255 (31.7%)
Administer to all cases in principle	69 (8.6%)
Do not administer in principle	31 (3.9%)
Not sure	55 (6.8%)
Q19: Do you administer PPI/P-CAB treatment to patients with reflux esophagitis after gastrectomy (excluding total gastrectomy)? (multiple answers allowed)—*n* (%)	
Administer if symptomatic	607 (75.5%)
Administer if LA grade A/B	117 (14.6%)
Administer if LA grade C/D	246 (30.6%)
Administer to all cases in principle	26 (3.2%)
Do not administer in principle	126 (15.7%)
Not sure	20 (2.5%)

*NBI* narrow-band imaging, *BLI* blue-laser imaging, *TXI* texture- and color-enhancement imaging, *LCI* linked color imaging, *PPI* proton pump inhibitor, *P-CAB* potassium-competitive acid blocker, *LA grade* Los Angeles grade

### Relevant landmarks in BE diagnosis (questions 5 and 6)

As indicated in Table 2, the majority of endoscopists used the distal end of the palisade vessels to determine the esophagogastric junction (EGJ) under both sedation and non-sedation conditions, with 47.4% using this landmark under sedation and 61.9% without sedation. In contrast, the minority of endoscopists relied on the proximal end of the gastric folds, with 13.1% using this landmark under sedation and 9.3% without sedation. Roughly 30% of endoscopists reported using both landmarks regardless of sedation status.

### Biopsy for BE diagnosis (questions 7 and 8)

The vast majority of Japanese endoscopists did not perform biopsies to detect intestinal metaplasia for the definitive diagnosis of BE (94.0%), nor did they perform random biopsies according to the Seattle protocol (93.0%), both of which are common practices in Western countries (Table 2). In addition, 6.2% of respondents described performing random biopsies based on the length of BE.

### Examination for LSBE (questions 9 and 10)

When performing endoscopy on patients with LSBE, over half of endoscopists (51.4%) described using a magnifying endoscope, while 28.2% and 20.4% reported using a non-magnifying endoscope or ultra-thin endoscope, respectively (Table 2). Multivariate analysis on the use of a magnifying endoscope demonstrated that work location area, proportion of endoscopic practice within total workload, membership in the JES, and primary work facility were the main contributing factors (Supplementary table S3). A large majority of endoscopists (93.9%) cited primarily employing narrow-band imaging (NBI) or blue-laser imaging (BLI).

### Diagnostic threshold and patient engagement (questions 11–13)

As indicated in Table 2, more than half of endoscopists diagnosed BE in the presence of any columnar epithelium, including less than 1 cm, at the distal end of the esophagus (53.6%), with the remaining respondents diagnosing BE only when the columnar epithelium was 1 cm or more. When those who did not select “All lengths, including < 1 cm” or “Not sure” for question 11 were asked why they did not diagnose BE for columnar epithelium of less than 1 cm (multiple answers allowed), the majority of endoscopists answered “Too many patients meet the criteria” (62.1%), followed next by “Low cancer risk for < 1 cm” (37.6%), “ < 1 cm is considered normal” (34.8%), and “Avoid causing unnecessary anxiety” (23.9%). In response to the question on whether they informed patients of the presence of BE, 30.7% of respondents cited that they told their patients even when the columnar epithelium was less than 1 cm.

### BE surveillance (questions 14–16)

Regarding questions on surveillance intervals for BE, responses were nearly evenly split for ultra-short segment BE (USSBE) of less than 1 cm, with 43.3% of endoscopists indicating “Do not recommend special surveillance” and 42.2% recommending surveillance every year (Table 2). For short-segment BE (SSBE) between 1 and 3 cm, roughly two-thirds of respondents advocated annual surveillance (65.7%), while 18.8% indicated that they did not recommend special surveillance. Approximately three-quarters of respondents recommended annual surveillance for LSBE of 3 cm or more, with 18.4% advising surveillance every 6 months.

### Drug administration (questions 17–19)

As shown in Table 2 regarding the question of whether to administer a proton pump inhibitor (PPI) or potassium-competitive acid blocker (P-CAB) upon diagnosing BE (multiple answers allowed), the majority of physicians responded that they administered these medications if the patient was symptomatic (74.8%), followed next by the presence of reflux esophagitis (52.9%). We observed a 6.3% response rate for administering PPI/P-CAB in cases of BE of 3 cm or more.

Following subtotal esophagectomy, the most common responses were PPI/P-CAB administration if the patient was symptomatic (77.9%) or had severe reflux esophagitis (Los Angeles classification [9] Grade C/D) (31.7%) (Table 2). In addition, 8.6% of respondents indicated they administered these drugs in all cases in principle. Similarly for patients receiving gastrectomy (excluding total gastrectomy), most endoscopists answered that they administered PPI/P-CAB for symptomatic cases (75.5%) and severe reflux esophagitis (30.6%), although the percentage of physicians who generally administered these medications decreased to 3.2%.

## Discussion

This first nationwide survey of BE management in Japan revealed several distinct gaps between Western EBM and Japanese practices. The key differences included low adherence to standardized inspection times, limited use of the Prague classification, and a preference for targeted over random biopsies.

### Observation time and documentation of BE (questions 3 and 4)

Endoscopic observation time and standardized documentation of BE length are essential for accurate diagnosis and surveillance. Longer observation times are associated with higher detection rates of HGD/EAC [8]. Accordingly, the European Society of Gastrointestinal Endoscopy (ESGE) Guidelines recommend a minimum of 1 min per centimeter of BE length during surveillance [6]. The Prague classification provides a standardized, validated method to report the circumferential (*C*) and maximal (*M*) extent of BE, which help determines cancer risk stratification and surveillance intervals [7]. Adherence to this system significantly improves dysplasia detection rates [9], and many guidelines recommend its use [4–6].

Our survey revealed notable differences between endoscopist adherence to Western and Japanese practices. Only 7.6% of physicians followed ESGE-recommended observation times, and 30% followed the Prague classification. Subgroup analysis showed higher adherence to established guidelines among JES members and physicians at high-volume institutions, suggesting that specialists of EAC dedicated more observation time to enhance early detection. The predominance of USSBE and SSBE in Japan likely contributed to the perceived lack of necessity for detailed observation and documentation. The absence of these aspects in Japanese guidelines may have also been a contributing factor. However, since BE length correlates with cancer risk, accurate documentation is indeed essential for proper risk stratification. These findings emphasize the need for further research and standardized recommendations regarding sufficient endoscopic observation time and precise documentation of BE length in future Japanese guidelines.

### Relevant landmarks in BE diagnosis (questions 5 and 6)

Most Society guidelines recommend the proximal end of the gastric folds as the landmark for identifying the EGJ [3, 5, 6]. In contrast, Japanese classification designates the distal end of the palisade vessels [10]. Whereas the proximal end of the gastric folds can shift with insufflation, the distal end of the palisade vessels may become indistinct due to inflammation or inadequate insufflation, indicating that both landmarks have advantages and limitations [11, 12].

Many participating physicians followed Japanese guidelines and frequently used the distal end of the palisade vessels as the EGJ landmark, regardless of sedation. This was likely due, in part, to the lower prevalence of severe reflux esophagitis and LSBE in Japan than in Western countries. A higher proportion of physicians used the palisade vessels under non-sedation, probably owing to improved esophageal wall distension from deep breathing, which facilitates observation.

### Biopsy for BE diagnosis (questions 7 and 8)

The presence of intestinal metaplasia in columnar-lined epithelium is widely recognized in Western countries as increasing carcinogenic potential [13]. As such, many guidelines require proof of intestinal metaplasia for a BE diagnosis [4–6]. No evidence supports this association in the Japanese population, and so national guidelines do not include this requirement [14]. Meanwhile, despite Western guidelines recommending random biopsies as outlined in the Seattle protocol [3–6], compliance is often poor in practice, particularly as BE length increases [15]. A meta-analysis demonstrated that targeted biopsies using NBI provided superior detection rates, suggestive of an alternative approach [16].

Our survey results showed that biopsy practices in Japan differed significantly from those in Western countries. Most Japanese endoscopists neither performed biopsies to confirm intestinal metaplasia nor adhered to random biopsy protocols. Instead, they relied on targeted biopsies using such imaging technologies as image-enhanced endoscopy (IEE) and magnifying endoscopy. This approach may reflect the lower prevalence of LSBE in Japan. As Western countries increasingly acknowledge the limitations of the Seattle protocol, Japanese practices may be more closely aligned with global trends favoring advanced imaging techniques for more precise surveillance.

### Examination for LSBE (questions 9 and 10)

Numerous studies have highlighted the utility of magnifying endoscopy, primarily for lesion characterization. When combined with such IEE techniques as NBI, however, magnifying endoscopy was found to improve the detection of early neoplasia in BE as well [17, 18]. In contrast, while ultra-thin endoscopes are effective for detecting BE, their role in tumor characterization remains limited [19]. Japanese guidelines recommend image-enhanced magnifying endoscopy for assessing the lateral extent of superficial EAC before endoscopic resection [20]. Among IEE modalities, NBI provides the strongest evidence, with an American Society for Gastrointestinal Endoscopy meta-analysis confirming its diagnostic accuracy for guiding targeted biopsies during surveillance [21]. Although BLI has no specific studies regarding neoplasia detection, its similarity to NBI suggests a comparable efficacy. Evidence on texture- and color-enhancement imaging (TXI) and linked color imaging (LCI) remains limited, and these modalities are not currently recommended in Japanese or Western guidelines.

Despite its proven benefits, only 51.4% of respondents cited using magnifying endoscopy for LSBE, with nearly half preferring non-magnifying or ultra-thin endoscopes. Subgroup analysis revealed higher usage among physicians dedicating more time to endoscopy and JES members, reflecting a greater awareness of LSBE risk and the modality’s advantages. The lower usage rate among clinic-based endoscopists was likely indicative of equipment limitations and consideration of patient throughput and comfort. The regional differences observed for usage rates highlighted a potential positive influence of local clinical leaders. However, while 93.9% of respondents used NBI or BLI for LSBE, thus demonstrating effective evidence integration, the low utilization of magnifying endoscopy in high-risk EAC patients underscored the need for further education and advocacy to emphasize the modality’s critical role in early detection. In facilities where magnifying endoscopy is not available, referring LSBE patients to high-volume centers with expertise in magnifying endoscopy may be advisable for a more comprehensive diagnosis.

### Diagnostic threshold and patient engagement (questions 11–13)

Western guidelines define BE as columnar epithelium extending at least 1 cm above the EGJ [5, 6], while Japanese guidelines judge BE as columnar epithelium continuous with the gastric mucosa without specifying a minimum length [14]. A nationwide Japanese study reported the annual EAC incidence of USSBE of less than 1 cm as extremely low at 0.0032% (0.00066–0.013%) per year [22]. Despite this very low risk, diagnosing BE, particularly USSBE, can cause disproportionate cancer-related anxiety in patients [23]. This difference in classification likely arose from the fact that BE in Japan was originally defined based on anatomic continuity rather than cancer risk, as evidence on BE length and EAC risk had been lacking. With more data supporting a correlation between BE length and EAC risk in Japan as in Western populations, reconsideration of the classification criteria is warranted.

Approximately half of respondents diagnosed columnar epithelium of less than 1 cm as BE, which was consistent with Japanese guidelines. However, the absence of the other half raises questions about whether this constitutes a standardized “definition.” Among those diagnosing USSBE as BE, 40% (n = 184) did not inform the diagnosis to patients, likely to avoid anxiety or due to low cancer risk. This tendency was more frequently observed among JES members (data not shown), suggesting that specialists were more likely to diagnose USSBE but withhold this information, while non-specialists tended to both diagnose USSBE and inform patients in adherence to guidelines. Our findings imply the need for careful discussion on whether to further enforce the current Japanese guidelines or consider revising them.

### BE surveillance (questions 14–16)

Western guidelines emphasize random biopsies to guide BE management and surveillance. Due to limited evidence on the surveillance intervals for non-dysplastic BE, some guidelines specify intervals [4, 6] while others do not [3, 5]. ESGE guidelines recommend no surveillance for USSBE of less than 1 cm, every 5 years for SSBE (1–3 cm), every 3 years for LSBE (3–10 cm), and referral to expert centers for BE exceeding 10 cm [6]. In contrast, Japanese guidelines suggest surveillance for BE of over 3 cm without specifying an interval and does not advocate monitoring for BE of under 3 cm due to insufficient evidence [14].

Approximately 40% of respondents recommended annual surveillance for USSBE in spite of its low cancer risk, which suggested excessive follow-up. A similar trend was observed for SSBE, likely owing to the easy access to endoscopy in Japan and differences in healthcare reimbursement compared with Western countries. Although Japanese patients have been proposed to have lower cancer risk, recent studies indicate that SSBE- and LSBE-related cancer incidences may be comparable to Western data [22]. Frequent surveillance in Japan may have contributed to increased cancer detection, but also highlights the lack of clear guidance on follow-up intervals in Japanese guidelines.

### Drug administration (questions 17–19)

The use of PPIs as chemoprevention for BE remains controversial. A meta-analysis reported a 71% reduction in HGD/EAC incidence with PPI use [24], which inferred a protective effect. However, the randomized prospective AspECT study found no definitive evidence supporting high-dose PPI or aspirin for cancer prevention [25]. Indeed, concerns about PPI side effects [26] have led many guidelines to recommend regular-dose PPI therapy [4–6], while Japanese guidelines do not advise PPI use for chemoprevention due to inadequate evidence [14].

The pathophysiology of postoperative esophagitis varies by surgical procedure. PPIs have shown particular efficacy after subtotal esophagectomy or proximal gastrectomy, where esophagitis rates are high [27, 28]. Japanese guidelines recognize the benefits of PPI therapy but provide no clear recommendations on timing or duration [29].

In line with Japanese guidelines, many physicians in our survey prescribed PPI/P-CAB for BE based on symptoms or endoscopic findings, and not for chemoprevention. Similarly, postoperative esophagitis treatment followed symptom-based decisions, generally reflecting guideline adherence. Routine PPI/P-CAB use was more common after subtotal esophagectomy than gastrectomy, which was likely influenced by differences in reconstruction methods.

### Future perspectives

This survey underscores the need to address gaps in BE management practices in Japan by focusing on key areas where evidence-based approaches remain underutilized. For instance, magnifying endoscopy for LSBE, which plays a critical role in early EAC detection and characterization, was used by only half of respondents and represents an opportunity for broader adoption. Our survey also highlighted the importance of developing Japan-specific evidence to address unique challenges, such as management for USSBE and defining surveillance intervals tailored to BE length. By integrating these findings into future guidelines and targeted dissemination efforts, Japan may better align its clinical practices with both international standards and domestic clinical realities.

### Limitations

This study had several limitations. First, the questionnaire format captured reported practices rather than actual clinical care, potentially introducing reporting bias. Moreover, voluntary participation might have skewed the results, reflecting practices from physicians with a particular interest in BE and EAC rather than the broader medical community. In addition, as the total number of distributed questionnaires was unknown, the response rate could not be calculated. This limitation may introduce response bias since the perspectives of the non-respondents remain uncertain. However, full disclosure of the respondents’ characteristics has been provided to enhance transparency on the representativeness of our sample. Moreover, the nationwide nature of this survey may provide a basis for the revision of diagnosis and surveillance guidelines for BE in Japan.

## Conclusion

This survey revealed significant gaps between EBM and real-world practice among Japanese clinicians, as well as differences in their adoption of Western and Japanese approaches. To optimize clinical practice for Japanese BE patients, further generation of local evidence, refinement of guidelines, and then wide dissemination of these updates among endoscopists are essential.

## Supplementary Information

Below is the link to the electronic supplementary material.

Supplementary file1 (DOCX 50 KB)

## Data Availability

The datasets analyzed during the current study are available from the corresponding author on reasonable request.
