# Supplementary material for: Evaluating the discrepancies between evidence-based and community standard practices in the endoscopic examination of Barrett’s esophagus: a nationwide survey in Japan
Source: Esophagus. 2025 Apr 19;22(3):349–59. doi: 10.1007/s10388-025-01127-6 (PMC12167344; doi:10.1007/s10388-025-01127-6)
Supplement: Supplementary file 1 — Supplementary file1 (DOCX 50 KB) [file 10388_2025_1127_MOESM1_ESM.docx]

| Supplementary table S1:  Multivariate analysis of Q3: “When performing endoscopy on patients with long-segment Barrett’s esophagus (LSBE) of 3 cm or more in maximum length, how much time do you spend based on the length of the Barrett’s segment?” Odds ratio for “More than 1 min per 1cm” | | | | |
| --- | --- | --- | --- | --- |
|  | Multivariate analysis | | | |
|  | More than 1 min per 1cm | Less than 1 min per 1 cm, or Not paying much attention | OR (95% CI) | *p* value |
| Age- no. (%) |  |  | Excluded from final model |  |
| -30 years | 0 (0.0) | 22 (100.0) |  |  |
| 31-40 years | 11 (6.1) | 170 (93.9) |  |  |
| 41-50 years | 19 (6.9) | 256 (93.1) |  |  |
| 51-60 years | 19 (9.8) | 175 (90.2) |  |  |
| 61-70 years | 9 (8.2) | 101 (91.8) |  |  |
| 71- years | 3 (13.6) | 19 (86.4) |  |  |
| Sex - no. (%) |  |  | Excluded from final model |  |
| Female | 8 (7.0) | 107 (93.0) |  |  |
| Male | 53 (7.7) | 636 (92.3) |  |  |
| Work location area - no. (%) | | | Excluded from final model |  |
| Hokkaido | 0 (0.0) | 10 (100.0) |  |  |
| Tohoku | 10 (4.9) | 195 (95.1) |  |  |
| Kanto | 24 (11.5) | 184 (88.5) |  |  |
| Chubu | 9 (7.4) | 113 (92.6) |  |  |
| Kinki | 9 (9.7) | 84 (90.3) |  |  |
| Chugoku-Shikoku | 6 (6.6) | 85 (93.4) |  |  |
| Kyushu | 3 (4.0) | 72 (96.0) |  |  |
| Specialty—no. (%) |  |  | Excluded from final model |  |
| Gastroenterology | 51 (7.7) | 610 (92.3) |  |  |
| Gastroenterological surgery | 8 (7.5) | 99 (92.5) |  |  |
| Others | 2 (5.6) | 34 (94.4) |  |  |
| The proportion of endoscopic practice within the total workload- no. (%) | | | Excluded from final model |  |
| 0-24% | 14 (6.9) | 188 (93.1) |  |  |
| 25-49% | 12 (5.7) | 198 (94.3) |  |  |
| 50-74% | 19 (8.0) | 218 (92.0) |  |  |
| 75-100% | 16 (10.3) | 139 (89.7) |  |  |
| Member of the Japan esophageal society - no. (%) | | |  |  |
| Yes | 37 (15.0) | 209 (85.0) | 4.34 (2.44-7.75) | <0.01 |
| No | 24 (4.3) | 534 (95.7) | 1 (Ref) |  |
| Japanese esophageal society esophagologist certification- no. (%) | | | Excluded from final model |  |
| Yes | 16 (13.7) | 101 (86.3) |  |  |
| No | 45 (6.6) | 642 (93.4) |  |  |
| Board certified fellow or trainer of the JGES- no. (%) | | | Excluded from final model |  |
| Yes | 51 (7.8) | 606 (92.2) |  |  |
| No | 10 (6.8) | 137 (93.2) |  |  |
| Primary work facility- no. (%) | | |  |  |
| University hospital | 20 (8.7) | 211 (91.3) | 1.03 (0.32-3.31) | 0.97 |
| Cancer specialty hospital | 4 (8.9) | 41 (91.1) | 1 (Ref) |  |
| Other hospitals | 22 (6.0) | 343 (94.0) | 1.84 (0.52-6.46) | 0.34 |
| Clinics | 15 (9.2) | 148 (90.8) | 3.90 (1.01-15.1) | 0.04 |
| Main purpose of endoscopic examinations you conduct - no. (%) | | | Excluded from final model |  |
| Regular medical practice | 55 (8.3) | 609 (91.7) |  |  |
| Health checkups and screenings | 6 (4.4) | 129 (95.6) |  |  |
| Others | 0 (0.0) | 5 (100.0) |  |  |
| Number of ESCC and EAC patients examined by endoscopy annually at the main work facility - no. (%) | | |  |  |
| Over 100 patients | 23 (13.8) | 144 (86.2) | 1 (Ref) |  |
| Under 100 patients | 38 (6.0) | 599 (94.0) | 0.31 (0.15-0.64) | <0.01 |

OR: Odds ratio, CI: Confidence interval, Ref: Reference, JGES: Japan Gastroenterological Endoscopy Society, ESCC: Esophageal squamous cell carcinoma, EAC: Esophageal adenocarcinoma

| Supplementary table S2:  Multivariate analysis of Q4: "When documenting the length of Barrett’s esophagus, do you use the Prague classification?" Odds ratio for “Yes” | | | | |
| --- | --- | --- | --- | --- |
|  | Multivariate analysis | | | |
|  | Yes | No, or Don’t know  about Prague  classification | OR (95% CI) | *p* value |
| Age- no. (%) |  |  | Excluded from final model |  |
| -30 years | 6 (27.3) | 16 (72.7) |  |  |
| 31-40 years | 71 (39.2) | 110 (60.8) |  |  |
| 41-50 years | 93 (33.8) | 182 (66.2) |  |  |
| 51-60 years | 82 (26.8) | 142 (73.2) |  |  |
| 61-70 years | 20 (18.2) | 90 (81.8) |  |  |
| 71- years | 5 (22.7) | 17 (77.3) |  |  |
| Sex - no. (%) |  |  | Excluded from final model |  |
| Female | 38 (33.0) | 77 (67.0) |  |  |
| Male | 209 (30.3) | 480 (69.7) |  |  |
| Work location area - no. (%) | | |  |  |
| Hokkaido | 4 (40.0) | 6 (60.0) | 1.85 (0.47-7.25) | 0.38 |
| Tohoku | 40 (19.5) | 165 (80.5) | 0.61 (0.36-1.02) | 0.06 |
| Kanto | 59 (28.4) | 149 (71.6) | 1 (Ref) |  |
| Chubu（ | 52 (42.6) | 70 (57.4) | 1.61 (0.95-2.74) | 0.08 |
| Kinki | 39 (41.9) | 54 (58.1) | 1.17 (0.66-2.08) | 0.60 |
| Chugoku-Shikoku | 29 (31.9) | 62 (68.1) | 0.98 (0.54-1.78) | 0.95 |
| Kyushu | 24 (32.0) | 51 (68.0) | 1.05 (0.56-1.97) | 0.89 |
| Specialty—no. (%) |  |  |  |  |
| Gastroenterology | 221 (33.4) | 440 (66.6) | 1.32 (0.54-3.21) | 0.54 |
| Gastroenterological surgery | 19 (17.8) | 88 (82.2) | 0.20 (0.07-0.58) | <0.01 |
| Others | 7 (19.4) | 29 (80.6) | 1 (Ref) |  |
| The proportion of endoscopic practice within the total workload- no. (%) | | | Excluded from final model |  |
| 0-24% | 34 (16.8) | 168 (83.2) |  |  |
| 25-49% | 57 (27.1) | 153 (72.9) |  |  |
| 50-74% | 90 (38.0) | 147 (62.0) |  |  |
| 75-100% | 66 (42.6) | 89 (57.4) |  |  |
| Member of the Japan esophageal society - no. (%) | | |  |  |
| Yes | 109 (44.3) | 137 (55.7) | 3.23 (2.14-4.87) | <0.01 |
| No | 138 (24.7) | 420 (75.3) | 1 (Ref) |  |
| Japanese esophageal society esophagologist certification- no. (%) | | | Excluded from final model |  |
| Yes | 47 (40.2) | 70 (59.8) |  |  |
| No | 200 (29.1) | 487 (70.9) |  |  |
| Board certified fellow or trainer of the JGES- no. (%) | | | Excluded from final model |  |
| Yes | 213 (32.4) | 444 (67.6) |  |  |
| No | 34 (23.1) | 113 (76.9) |  |  |
| Primary work facility- no. (%) | | |  |  |
| University hospital | 99 (42.9) | 132 (57.1) | 1.23 (0.60-2.54) | 0.57 |
| Cancer specialty hospital | 22 (48.9) | 23 (51.1) | 1 (Ref) |  |
| Other hospitals | 104 (28.5) | 261 (71.5) | 0.96 (0.46-1.99) | 0.90 |
| Clinics | 22 (13.5) | 141 (86.5) | 0.44 (0.80-1.01) | 0.05 |
| Main purpose of endoscopic examinations you conduct - no. (%) | | | Excluded from final model |  |
| Regular medical practice | 222 (33.4) | 442 (66.6) |  |  |
| Health checkups and screenings | 25 (18.5) | 110 (81.5) |  |  |
| Others | 0 (0.0) | 5 (100.0) |  |  |
| Number of ESCC and EAC patients examined by endoscopy annually at the main work facility - no. (%) | | |  |  |
| Over 100 patients | 87 (52.1) | 80 (47.9) | 1 (Ref) |  |
| Under 100 patients | 160 (25.1) | 477 (74.9) | 0.48 (0.31-0.75) | <0.01 |

OR: Odds ratio, CI: Confidence interval, Ref: Reference, JGES: Japan Gastroenterological Endoscopy Society, ESCC: Esophageal squamous cell carcinoma, EAC: Esophageal adenocarcinoma

|  |
| --- |

| Supplementary table S3:  Multivariate analysis of Q9: "When performing endoscopy on a patient with known long-segment Barrett’s esophagus (LSBE), which endoscope do you most frequently use?" Odds ratio for “Magnifying endoscope” | | | | |
| --- | --- | --- | --- | --- |
|  | Multivariate analysis | | | |
|  | Magnifying endoscope | Non-magnifying  endoscope, or  Ultra-thin endoscope | OR (95% CI) | *p* value |
| Age- no. (%) |  |  | Excluded from final model |  |
| -30 years | 14 (63.6) | 8 (36.4) |  |  |
| 31-40 years | 120 (66.3) | 61 (33.7) |  |  |
| 41-50 years | 139 (50.5) | 136 (49.5) |  |  |
| 51-60 years | 98 (50.5) | 96 (49.5) |  |  |
| 61-70 years | 37 (33.6) | 73 (66.4) |  |  |
| 71- years | 5 (22.7) | 17 (77.3) |  |  |
| Sex - no. (%) |  |  | Excluded from final model |  |
| Female | 53 (46.1) | 62 (53.9) |  |  |
| Male | 360 (52.2) | 329 (47.8) |  |  |
| Work location area - no. (%) | | |  |  |
| Hokkaido | 2 (20.0) | 8 (80.0) | 0.15 (0.03-0.83) | 0.03 |
| Tohoku | 80 (39.0) | 125 (61.0) | 0.60 (0.36-1.01) | 0.05 |
| Kanto | 89 (42.8) | 119 (57.2) | 1 (Ref) |  |
| Chubu | 80 (65.6) | 42 (34.4) | 1.84 (1.03-3.30) | 0.04 |
| Kinki | 62 (66.7) | 31 (33.3) | 1.38 (0.73-2.59) | 0.32 |
| Chugoku-Shikoku | 51 (56.0) | 40 (44.0) | 1.35 (0.73-2.50) | 0.35 |
| Kyushu | 49 (65.3) | 26 (34.7) | 2.35 (1.20-4.59) | 0.01 |
| Specialty—no. (%) |  |  |  |  |
| Gastroenterology | 373 (56.4) | 288 (43.6) | 2.37 (0.86-6.51) | 0.09 |
| Gastroenterological surgery | 34 (31.8) | 73 (68.2) | 0.55 (0.17-1.71) | 0.30 |
| Others | 6 (16.7) | 30 (83.3) | 1 (Ref) |  |
| The proportion of endoscopic practice within the total workload- no. (%) | | |  |  |
| 0-24% | 58 (28.7) | 144 (71.3) | 1 (Ref) |  |
| 25-49% | 91 (43.3) | 119 (56.7) | 1.27 (0.77-2.08) | 0.44 |
| 50-74% | 156 (65.8) | 81 (34.2) | 2.30 (1.36-3.90) | <0.01 |
| 75-100% | 108 (69.7) | 47 (30.3) | 2.45 (1.37-4.37) | <0.01 |
| Member of the Japan esophageal society - no. (%) | | |  |  |
| Yes | 151 (61.4) | 95 (38.6) | 2.48 (1.52-4.05) | <0.01 |
| No | 262 (47.0) | 296 (53.0) | 1 (Ref) |  |
| Japanese esophageal society esophagologist certification- no. (%) | | | Excluded from final model |  |
| Yes | 64 (54.7) | 53 (45.3) |  |  |
| No | 349 (50.8) | 338 (49.2) |  |  |
| Board certified fellow or trainer of the JGES- no. (%) | | | Excluded from final model |  |
| Yes | 341 (51.9) | 316 (48.1) |  |  |
| No | 72 (49.0) | 75 (51.0) |  |  |
| Primary work facility- no. (%) | | |  |  |
| University hospital | 150 (64.9) | 81 (35.1) | 0.70 (0.29-1.72) | 0.44 |
| Cancer specialty hospital | 37 (82.2) | 8 (17.8) | 1 (Ref) |  |
| Other hospitals | 196 (53.7) | 169 (46.3) | 0.62 (0.26-1.47) | 0.28 |
| Clinics | 30 (18.4) | 133 (81.6) | 0.12 (0.05-0.30) | <0.01 |
| Main purpose of endoscopic examinations you conduct - no. (%) | | |  |  |
| Regular medical practice | 385 (58.0) | 279 (42.0) | 0.77 (0.08-6.94) | 0.81 |
| Health checkups and screenings | 25 (18.5) | 110 (81.5) | 0.12 (0.01-1.10) | 0.06 |
| Others | 3 (60.0) | 2 (40.0) | 1 (Ref) |  |
| Number of ESCC and EAC patients examined by endoscopy annually at the main work facility - no. (%) | | | Excluded from final model |  |
| Over 100 patients | 121 (72.5) | 46 (27.5) |  |  |
| Under 100 patients | 292 (45.8) | 345 (54.2) |  |  |

OR: Odds ratio, CI: Confidence interval, Ref: Reference, JGES: Japan Gastroenterological Endoscopy Society, ESCC: Esophageal squamous cell carcinoma, EAC: Esophageal adenocarcinoma
